# Role of blood gas analysis during cardiopulmonary resuscitation in out-of-hospital cardiac arrest patients

**DOI:** 10.1097/MD.0000000000003960

**Published:** 2016-06-24

**Authors:** Youn-Jung Kim, You Jin Lee, Seung Mok Ryoo, Chang Hwan Sohn, Shin Ahn, Dong-Woo Seo, Kyoung Soo Lim, Won Young Kim

**Affiliations:** Department of Emergency Medicine, University of Ulsan College of Medicine, Asan Medical Centre, Seoul, Korea.

**Keywords:** blood gas analysis, cardiopulmonary resuscitation, out-of-hospital cardiac arrest, prognosis

## Abstract

To determine the relationship between acid–base findings, such as pH, pCO_2_, and serum lactate levels, obtained immediately after starting cardiopulmonary resuscitation and the return of spontaneous circulation (ROSC).

A prospective observational study of adult, nontraumatic out-of-hospital cardiac arrest (OHCA) patients was conducted at an urban academic teaching institution between April 1, 2013 and March 31, 2015. Arterial blood sample for acid–base data was taken from all OHCA patients on arrival to the emergency department. Of 224 OHCA patients, 88 patients with unavailable blood samples or delayed blood sampling or ROSC within 4 minutes were excluded, leaving 136 patients for analysis.

The pH in the ROSC group was significantly higher than in the non-ROSC group (6.96 vs. 6.85; *P* *=* 0.009). pCO_2_ and lactate levels in the ROSC group were significantly lower than those in the non-ROSC group (74.0 vs. 89.5 mmHg, *P* *<* 0.009; 11.6 vs. 13.6 mmol/L, *P* *=* 0.044, respectively). In a multivariate regression analysis, pCO_2_ was the only independent biochemical predictor for sustained ROSC (OR 0.979; 95% CI 0.960–0.997; *P* *=* 0.025) and pCO_2_ of <75 mmHg was 3.3 times more likely to achieve ROSC (OR 0.302; 95% CI 0.146–0.627; *P* = 0.001).

pCO_2_ levels obtained during cardiopulmonary resuscitation on ER arrival was associated with ROSC in OHCA patients. It might be a potentially marker for reflecting the status of the ischemic insult. These preliminary results need to be confirmed in a larger population.

## 1 Introduction

Cardiac arrest and the consequent interruption of blood flow to metabolically active tissues cause intense hypercarbic and metabolic acidosis, resulting in the accumulation of end products, such as CO_2_, lactate, and hydrogen ions.^[1–3]^ Low flow time is a well-known predictor for outcome in out-of-hospital cardiac arrest (OHCA) patients; however, identifying the low flow time or no flow time is often impossible in unwitnessed patients. Additionally, even though the OHCA patients were witnessed and administered bystander CPR (cardiopulmonary resuscitation), the quality of CPR could be questionable. Even when conventional CPR is applied for cardiac arrest patients, it provides at best one-quarter of cardiac output and steadily degrades overtime.^[4,5]^ It may be important to perform initial blood gas analysis during CPR, as it can reflect the status of the ischemic insult on a cellular level. Blood gas analysis during CPR also might reflect the low flow time and it may be a tool to decide to escalate to more invasive strategies, such as extracorporeal CPR.^[6,7]^

In previous studies, emergency department (ED) presentation data, such as lactate, base excess, end-tidal carbon dioxide, potassium, and ammonia, have surfaced as potential predictors of outcome in OHCA.^[8–10]^ Specifically, low ammonia level of 170 μg/dL and lactate level of <12.0 mmol/L were prognostic factors for favorable outcome.^[10]^ However, these variables have been evaluated using data after sustained ROSC, not during CPR or not available at the point of time.^[11,12]^ The acid–base findings upon arrival to the ED have not been sufficiently evaluated. We hypothesized that blood gas analysis during CPR might be a good tool to measure ischemic insult on a cellular level and may be associated with outcome in patients with OHCA. Therefore, the aims of this study were to describe the acid–base findings of OHCA patients obtained immediately after starting CPR and determine which acid–base findings are useful in predicting sustained ROSC in OHCA patients.

## 2 Methods

### 2.1 Study design and population

This prospective observational study was conducted at the ED of a university-affiliated teaching hospital in Seoul, Korea, with an annual census of approximately 100,000 visits, between April 1, 2013 and March 31, 2015. In Korea, Emergency medical service (EMS) providers are encouraged to scoop and run to the ED while giving CPR during ambulance transport as soon as possible after giving 1 cycle of CPR and EMS is not legally allowed to declare death in the field except for obvious signs incompatible with life. EMS providers in Korea are trained in Basic Life Support to provide chest compressions and ventilation with bag-mask device and to apply an automated external defibrillator. Most of them are not qualified for advanced airway management as well as intravenous drug administration during CPR.

The study population consisted of all consecutive adult (age ≥18 years), nontraumatic OHCA patients. During the study period, a blood sample was taken from all OHCA patients within 4 minutes of arrival to the ED. Patients were excluded if the blood sample withdrawal was delayed (over 4 minutes), the blood sample was not obtained during CPR, they achieved sustained ROSC within 4 minutes, or they had a do-not-resuscitation order. Achievement of sustained ROSC was declared when patients had a palpable pulse for >20 minutes. Before commencing this study, the Institutional Review Board of the hospital approved our study and waived the requirement for informed consent.

### 2.2 Data

Demographic data were obtained from EMS reports and medical records. We extracted the following data: demographic characteristics, cause of cardiac arrest, electrocardiogram rhythm at the scene, bystander administration of CPR, prehospital resuscitation time, initial blood gas data within 4 minutes after ED arrival, and outcome (achievement of sustained ROSC). Basic life support and advanced cardiovascular life support were performed in accordance with the current Advanced Cardiac Life Support guidelines of 2010.^[13]^

Blood samples for blood gas analysis were collected from the radial or femoral artery using sodium-heparin-coated syringes. Blood gas analysis was performed using a GEM Premier 3000 (Instrumentation Laboratory, Lexington, MA). The detection ranges were as follows: blood pH, 6.80–7.80; pCO_2_, 5–115 mmHg; pO_2_, 0–760 mmHg; lactate, 0.3–15.0 mmol/L. For electrolytes, the detection ranges were as follows: sodium, 100–200 mmol/L; potassium, 0.1–20.0 mmol/L; glucose, 20–500 mg/dL. Test results were available within 85 seconds. In cases where the values exceeded their instrument limit, those values were assumed as the limit value.

### 2.3 Statistical analysis

Continuous variables are expressed as mean ± standard deviation (*SD*) when normally distributed and median with interquartile range (IQR) when non-normally distributed. Variables were tested for normal distribution using the Kolmogorov–Smirnov test. Categorical data are presented as absolute numbers and percent frequencies. The Student's *t*-test was used to compare the values of normally distributed continuous variables, and the Mann–Whitney *U* test was used to compare the values of non-normally distributed continuous variables. Differences between categorical variables were analyzed by the Chi-square test or the Fisher's exact test, as appropriate. We excluded the missing values in analysis and stated the proportion of the missing value in our manuscript. At baseline, clinically significant baseline characteristics and blood gas data as potential predictors of ROSC were first examined using univariate logistic analysis. Of the earlier reported variables, age, gender, pH, PCO_2_, and serum lactate level were candidates for the multivariable model and examined using multiple logistic regression analysis. The results of the multivariate logistic regression analyses were summarized by estimating the odds ratios (ORs) and the respective 95% confidence intervals (CIs). The Hosmer–Lemeshow test for logistic regression model was performed. The cut-off value of pCO_2_ for prediction of ROSC was calculated using a receiver operating characteristics (ROCs) curve and area under the ROC curve (AUC). A 2-tailed *P* value of <0.05 was considered statistically significant. All statistical analyses were performed with SPSS version 18.0 (IBM, Armonk, NY).

## 3 Results

**Figure 1:**
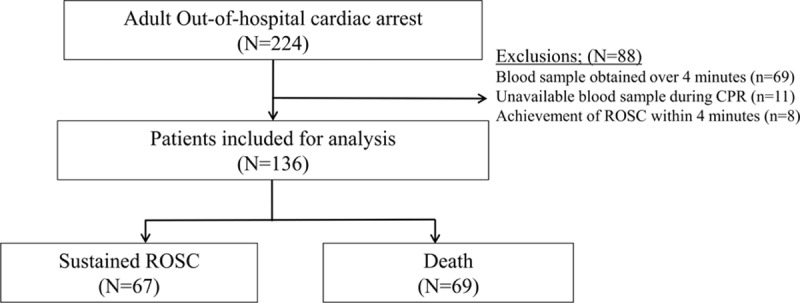
Cardiac arrest patients and study participants. ROSC = return of spontaneous circulation.

During the study period, 224 adult, nontraumatic cardiac arrest patients arrived in our ED. Of these, 88 patients were excluded for the following reasons: 69 patients had delayed blood sampling over 4 minutes, 11 patients were unavailable for blood sampling during CPR because of technical difficulties in vascular access, and 8 patients achieved ROSC within 4 minutes of ED arrival. This left 136 patients for analysis (Fig. 1). Sixty-seven patients (49.3%) achieved sustained ROSC, and 69 patients (50.7%) did not. One-fifth of the patients (22/136, 21.3%) survived at 1 month, and 7 of 136 patients (5.1%) had favorable neurological outcome with cerebral performance categories score of 1 or 2 at 1 month. Prehospital drug administration and advanced airway management were not conducted in all patients.

The biochemical parameters of our study patients, obtained within 4 minutes of ED arrival using point-of-care testing, are summarized in Table 1. The values from blood gas analysis were widely distributed and skewed to extreme values. Hypoxia (pO_2_, 17.5 mmHg, IQR, 9.0–45.8), hypercapnia (pCO_2_, 78.0 mmHg, IQR, 63.0–98.0), and increased lactate levels (12.5 mmol/L, IQR, 9.2–15.0) were evident, which contributed to the severe acidosis (pH, 6.89; IQR, 6.80–7.02) in the OHCA patients. Hyperkaliemia (potassium, 6.4mmol/L; IQR, 5.0–7.8) was a distinct electrolyte abnormality. Notably, the results of blood gas analysis for bicarbonate and base excess were technically unreadable for 43 patients (27 in the non-ROSC achievement group, 16 in the ROSC achievement group). In contrast, pH and lactate levels were available for all of the eligible OHCA patients. pCO_2_ levels were not available for 5 patients.

**Table 1:**
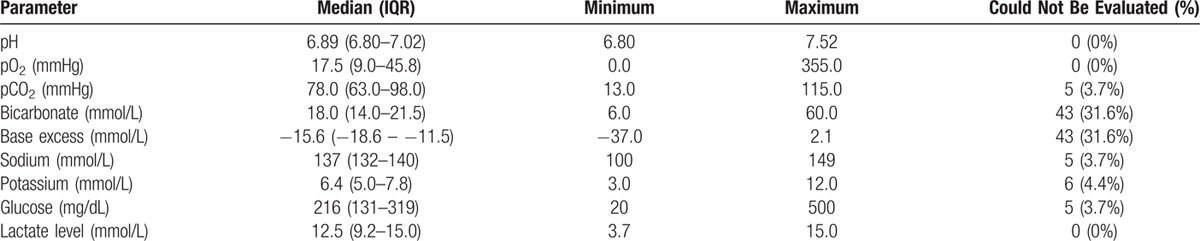
Blood Gas Analysis of Out-of-Hospital Cardiac Arrest Patients.

**Figure 2:**
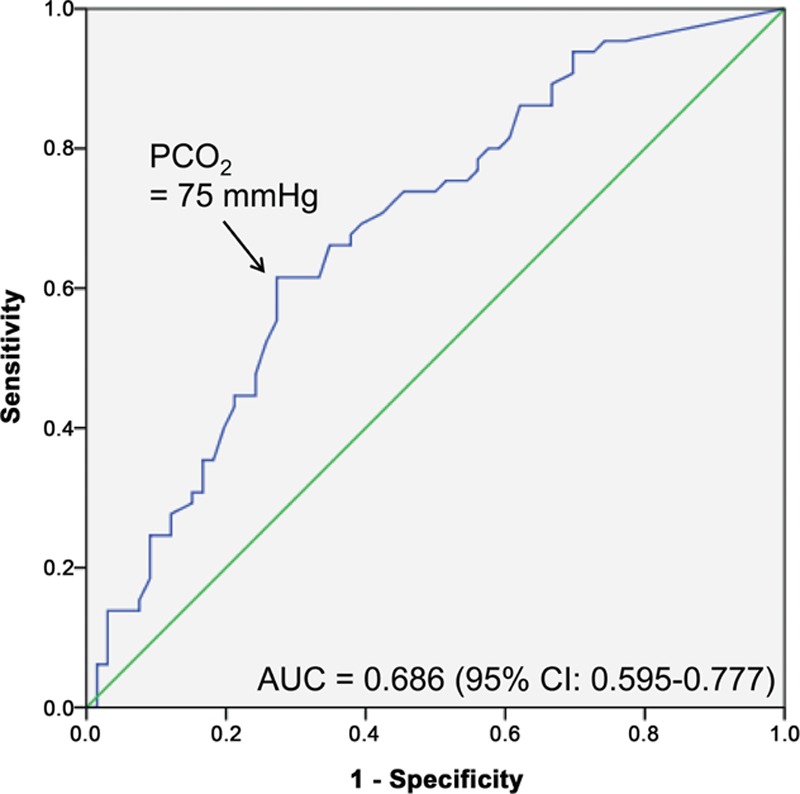
ROC curve of pCO_2_ for ROSC. ROSC = return of spontaneous circulation.

Table 2 lists the baseline characteristics and biochemical parameters of the non-ROSC achievement group and the ROSC achievement group. Statistically significant differences between these groups were evident in terms of gender, pH, pCO_2_, prehospital low flow time, and serum lactate levels. Of these 136 patients, 90 were male, and the median age was 67.5 years. The median time of prehospital low flow time (elapsed time to ED arrival after resuscitation) was 23.0 (16.5–29.0) minutes. The majority of our study patients (132/136, 97.1%) were intubated using endotracheal tube at ED admission while the last 4 patients were managed using supraglottic airway devices. Intubation and blood sampling were performed almost at the same time for our study patients. The median time to intubation was 2 minutes with IQR 1 to 3 minutes after ED arrival and the median time to blood sampling was 3 minutes with IQR 2 to 4 minutes. Blood sampling was performed before intubation in two-fifths of our patients (54/136, 39.7%). The pH result was less than the lower instrument limit of 6.8 for 47 patients (34.5% of total patients; 28 in the non-ROSC achievement group; 19 in the ROSC achievement group). Similarly, serum lactate levels for 40 patients exceeded the upper instrument limit of 15.0 mmol/L (29.4% of total patients; 22 in the non-ROSC achievement group, 18 in the ROSC achievement group). In contrast to pH and lactate levels, only 18 patients had pCO_2_ levels that exceeded the upper instrument limit of 115 mmHg (13.2% of total patients; 15 in the non-ROSC achievement group, 3 in the ROSC achievement group). pCO_2_ and lactate levels in the ROSC achievement group were significantly lower than those in the non-ROSC achievement group, whereas the pH in the ROSC achievement group was significantly higher than in the non-ROSC achievement group (*P* < 0.05; Table 2). Multivariate logistic regression analysis identified pCO_2_ as the only independent predictor of sustained ROSC in OHCA patients (OR: 0.979; 95% CI: 0.960–0.997; *P* = 0.025; Table 3). The AUC of pCO_2_ was 0.686 (95% CI: 0.595–0.777) for sustained ROSC (Fig. 2). By applying a cut-off value of PCO_2_ <75 mmHg, the possibility of the ROSC were 3.3 times more likely in patients below the cut-off level (OR: 0.302; 95% CI: 0.146–0.627; *P* = 0.001).

**Table 2:**
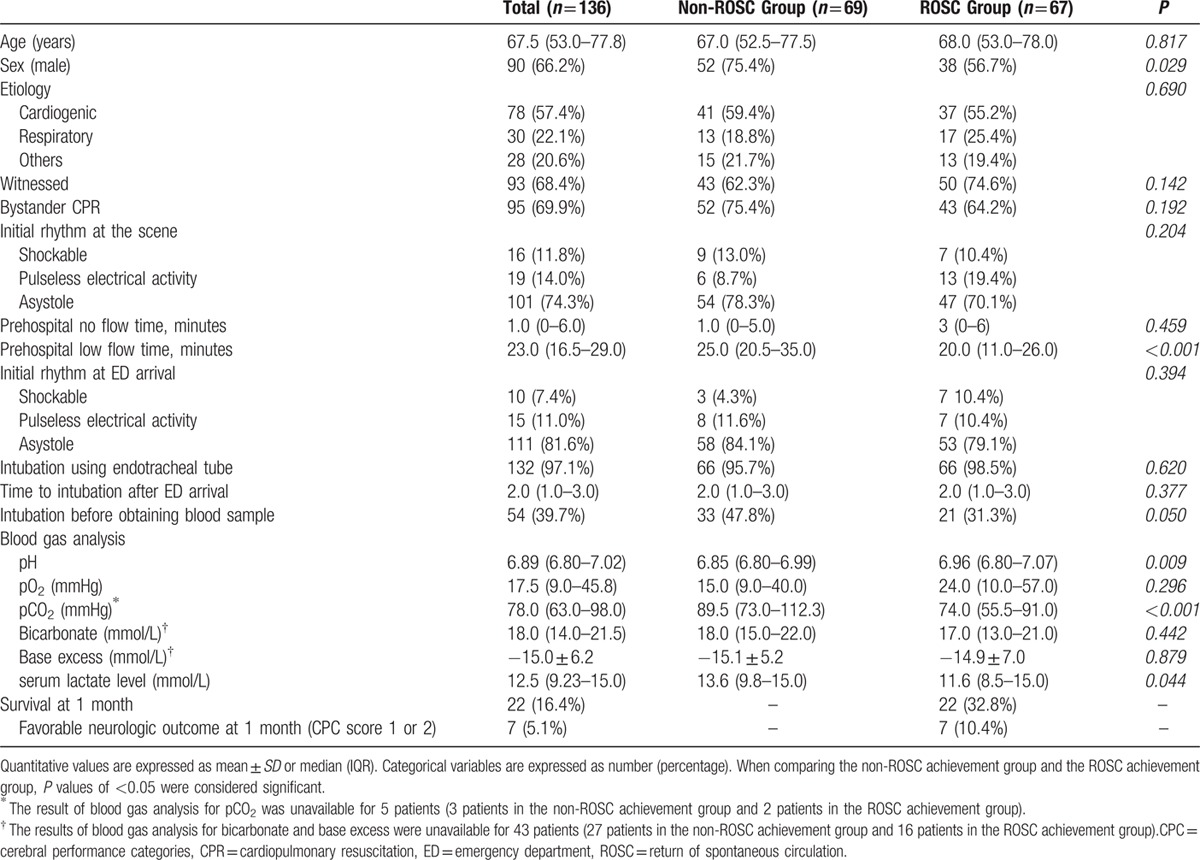
Characteristics and Biochemical Parameters of Out-of-Hospital Cardiac Arrest Patients.

**Table 3:**
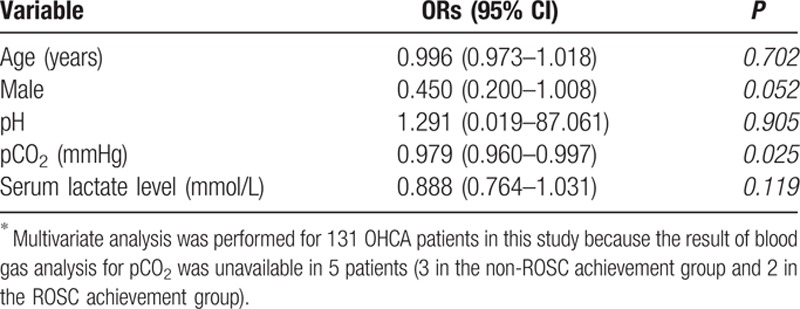
Multivariate Analysis for Factors Predicting Sustained Return of Spontaneous Circulation.^∗^

## 4 Discussion

In our current study, we demonstrated that hypoxemia, hypercarbic acidosis, and lactic acidosis were dominant blood gas abnormalities in OHCA patients immediately after ED arrival. Despite prehospital resuscitation, decreased ventilation and perfusion contributed to the progression of tissue ischemia. Among our abnormal findings, pCO_2_ level on ED arrival was found to be an independent predictor for sustained ROSC in OHCA patients.

Severe respiratory and metabolic acidosis as well as hyperkaliemia were obvious in many of our current study patients and were most likely caused by hypoperfusion and anaerobic metabolism in dysoxic tissue, which were more of a consequence than cause. However, acidosis and hyperkaliemia are treatable causes of cardiac arrest, especially in cases of pulseless electrical activity. Current guidelines emphasize identifying and treating the cause of cardiac arrest, which improves the survival rate.^[14]^ Although sodium bicarbonate administration is recommended for the treatment of severe/preexisting acidosis and hyperkaliemia, there are no current guidelines or suggestions regarding the levels of acidosis and potassium that require treatment. In our present study, the median pH was 6.89 (IQR, 6.80–7.02) in the OHCA patients, and 34.5% of our study patients had a pH below the lower instrument limit of 6.8. This result might suggest that acidosis measured within 4 minutes of CPR is not a reliable indicator of the severity of tissue hypoxemia because many patients had pH levels below the level of detection.

One of the novel findings in this study is that pCO_2_ was an independent predictor of sustained ROSC in OHCA patients, while other variables, such as lactate and pH were not. Animal models have shown a gradual increase of lactate during CPR,^[15–17]^ and the degree of lactate clearance was associated with the outcome of OHCA patients.^[17,18]^ However, the role of lactate had not been determined in clinical trials, especially in the early phase of CPR. The median lactate level was 12.5 mmol/L (IQR, 9.2–15.0), but, similarly to pH, the result of serum lactate for 40 patients (29.4%) exceeded the upper instrument limit of 15.0 mmol/L. This might imply that serum lactate during the early phase of CPR has a limitation as an indicator of the severity of tissue hypoxemia or downtime. In contrast to pH and lactate, only 18 patients (13.2%) had pCO_2_ levels that exceed the upper instrument limit of 115 mmHg, suggesting that pCO_2_ might be a reliable indicator of tissue hypoxemia and/or downtime. The possibility of achievement of ROSC was 3.3 times more likely when PCO_2_ was <75 mmHg and 89% of the ROSC achievement group had pCO_2_ levels <100 mmHg.

Recently, there has been increasing evidence that extracorporeal membrane oxygenation is an effective therapeutic option for OHCA patients who are refractory to classical resuscitation attempts.^[19,20]^ As the probability of survival with good neurologic outcome declined rapidly during CPR, early prediction of the probability of achieving sustained ROSC in OHCA patients is necessary for clinical decision-making.^[19–21]^ Although there is insufficient evidence on who is the right candidate for extracorporeal membrane oxygenation during CPR in OHCA patients, extracorporeal CPR may be considered for OHCA patients without irreversible ischemic brain damage. Although further studies will be needed to clarify the role of initial pCO_2_ in OHCA patients, initial pCO_2_ might be used as an indirect indicator which reflects the anoxic or hypoxic time in OHCA patients. A recent study has suggested that blood ammonia level (<84 μmol/L) on hospital arrival is useful in predicting non-ROSC with 94.5% sensitivity and 75.0% specificity.^[22]^ However, blood ammonia is not an appropriate laboratory test in this situation because it is time consuming. When an OHCA patient arrives at the ED, measuring pCO_2_ by blood gas analysis might help determine if more invasive strategies, such as extracorporeal CPR, are warranted.

The results of our study should be interpreted in the context of certain limitations. First, our study is from a single institution, which limits the generalization of our findings to other institutions or patients population and the sample size was relatively limited. Second, 88 of the initial patients were excluded (39.3%; 69 with delayed blood sampling over 4 minutes, 11 with unavailable blood sample due to technical difficulties in vascular access, and 8 that achieved ROSC within 4 minutes). Third, base excess and bicarbonate levels were uncheckable for 31.6% (43/136) of the patients who were included in our current analyses. Fourth, blood sampling during CPR is technically difficult, and it was difficult to distinguish if blood samples were obtained from an artery or a vein during CPR. Differences between arterial and venous pO_2_ and pCO_2_ levels have been documented.^[23]^ Additionally, we defined immediate arterial blood gas analysis as obtaining blood sample within 4 minutes after ED arrival. The definition was determined based on the data of our hospital, in which the median time was 3 minutes with IQR 2 to 4 minutes. Since there have been no studies that used initial arterial blood gas data during CPR, further research for optimal timing would be necessary. The EMS system in Korea differs to that of Western Europe and North America. The results can be influenced by the different local EMS systems with different protocol or algorithm for OHCA patients. Thus, our results should be interpreted with caution. Finally, the majority of our current study patients presented with a nonshockable rhythm at the scene and had >20 minutes of resuscitation time, which limits the generalization of our findings to other institutions or populations.

## 5 Conclusions

The pCO_2_ level obtained within 4 minutes of starting CPR is an independent predictor for sustained ROSC in OHCA patients, and it may provide the status of the ischemic insult because serum lactate and pH are unreliable when measured in this manner as it is frequently above the measurable limit. However, further studies will be needed to clarify the role of the blood gas analysis in clinical practice.

## References

[1] Prause G Ratzenhofer-Comenda B Smolle-Juttner F Comparison of lactate or BE during out-of-hospital cardiac arrest to determine metabolic acidosis. *Resuscitation* 2001; 51:297–300. 11738782 10.1016/s0300-9572(01)00424-5

[2] Takasu A Sakamoto T Okada Y Arterial base excess after CPR: the relationship to CPR duration and the characteristics related to outcome. *Resuscitation* 2007; 73:394–399. 17289244 10.1016/j.resuscitation.2006.10.014

[3] Ahn S Kim WY Sohn CH Seo DW Kim W Lim KS Potassium values in cardiac arrest patients measured with a point-of-care blood gas analyzer. *Resuscitation* 2011; 82:e25–e26. 21871855 10.1016/j.resuscitation.2011.08.010

[4] Bellamy RF DeGuzman LR Pedersen DC Coronary blood flow during cardiopulmonary resuscitation in swine. *Circulation* 1984; 69:174–180. 6689642 10.1161/01.cir.69.1.174

[5] Johnson BA Weil MH Redefining ischemia due to circulatory failure as dual defects of oxygen deficits and of carbon dioxide excesses. *Crit Care Med* 1991; 19:1432–1438. 1935165 10.1097/00003246-199111000-00021

[6] Kim WY Giberson TA Uber A Berg K Cocchi MN Donnino MW Neurologic outcome in comatose patients resuscitated from out-of-hospital cardiac arrest with prolonged downtime and treated with therapeutic hypothermia. *Resuscitation* 2014; 85:1042–1046. 24746783 10.1016/j.resuscitation.2014.04.005 PMC4087050

[7] Sawyer KN Kurz MC Caution when defining prolonged downtime in out of hospital cardiac arrest as extracorporeal cardiopulmonary resuscitation becomes accessible and feasible. *Resuscitation* 2014; 85:979–980. 24925119 10.1016/j.resuscitation.2014.05.018

[8] Sasson C Rogers MA Dahl J Kellermann AL Predictors of survival from out-of-hospital cardiac arrest: a systematic review and meta-analysis. *Circ Cardiovasc Qual Outcomes* 2010; 3:63–81. 20123673 10.1161/CIRCOUTCOMES.109.889576

[9] Grmec S Krizmaric M Mally S Kozelj A Spindler M Lesnik B Utstein style analysis of out-of-hospital cardiac arrest–bystander CPR and end expired carbon dioxide. *Resuscitation* 2007; 72:404–414. 17161518 10.1016/j.resuscitation.2006.07.012

[10] Shinozaki K Oda S Sadahiro T Blood ammonia and lactate levels on hospital arrival as a predictive biomarker in patients with out-of-hospital cardiac arrest. *Resuscitation* 2011; 82:404–409. 21227564 10.1016/j.resuscitation.2010.10.026

[11] Bender PR Debehnke DJ Swart GL Hall KN Serum potassium concentration as a predictor of resuscitation outcome in hypothermic cardiac arrest. *Wilderness Environ Med* 1995; 6:273–282. 11990091 10.1580/1080-6032(1995)006[0273:spcaap]2.3.co;2

[12] Levine RL Wayne MA Miller CC End-tidal carbon dioxide and outcome of out-of-hospital cardiac arrest. *N Engl J Med* 1997; 337:301–306. 9233867 10.1056/NEJM199707313370503

[13] Travers AH Rea TD Bobrow BJ Part 4: CPR overview: 2010 American Heart Association Guidelines for Cardiopulmonary Resuscitation and Emergency Cardiovascular Care. *Circulation* 2010; 122:S676–S684. 20956220 10.1161/CIRCULATIONAHA.110.970913

[14] Neumar RW Otto CW Link MS Part 8: adult advanced cardiovascular life support: 2010 American Heart Association Guidelines for Cardiopulmonary Resuscitation and Emergency Cardiovascular Care. *Circulation* 2010; 122:S729–S767. 20956224 10.1161/CIRCULATIONAHA.110.970988

[15] Hopper K Borchers A Epstein SE Acid base, electrolyte, glucose, and lactate values during cardiopulmonary resuscitation in dogs and cats. *J Vet Emerg Crit Care (San Antonio)* 2014; 24:208–214. 24739035 10.1111/vec.12151

[16] Cairns CB Niemann JT Pelikan PC Sharma J Ionized hypocalcemia during prolonged cardiac arrest and closed-chest CPR in a canine model. *Ann Emerg Med* 1991; 20:1178–1182. 1952301 10.1016/s0196-0644(05)81466-0

[17] Carden DL Martin GB Nowak RM Foreback CC Tomlanovich MC Lactic acidosis as a predictor of downtime during cardiopulmonary arrest in dogs. *Am J Emerg Med* 1985; 3:120–124. 3970767 10.1016/0735-6757(85)90033-6

[18] Donnino MW Miller J Goyal N Effective lactate clearance is associated with improved outcome in post-cardiac arrest patients. *Resuscitation* 2007; 75:229–234. 17583412 10.1016/j.resuscitation.2007.03.021

[19] Fagnoul D Combes A De Backer D Extracorporeal cardiopulmonary resuscitation. *Curr Opin Crit Care* 2014; 20:259–265. 24785674 10.1097/MCC.0000000000000098

[20] Wang CH Chou NK Becker LB Improved outcome of extracorporeal cardiopulmonary resuscitation for out-of-hospital cardiac arrest–a comparison with that for extracorporeal rescue for in-hospital cardiac arrest. *Resuscitation* 2014; 85:1219–1224. 24992872 10.1016/j.resuscitation.2014.06.022

[21] Reynolds JC Frisch A Rittenberger JC Callaway CW Duration of resuscitation efforts and functional outcome after out-of-hospital cardiac arrest: when should we change to novel therapies? *Circulation* 2013; 128:2488–2494. 24243885 10.1161/CIRCULATIONAHA.113.002408 PMC4004337

[22] Lin CH Chi CH Wu SY Prognostic values of blood ammonia and partial pressure of ammonia on hospital arrival in out-of-hospital cardiac arrests. *Am J Emerg Med* 2013; 31:8–15. 22795429 10.1016/j.ajem.2012.04.037

[23] Steedman DJ Robertson CE Acid base changes in arterial and central venous blood during cardiopulmonary resuscitation. *Arch Emerg Med* 1992; 9:169–176. 1326975 10.1136/emj.9.2.169 PMC1285855

