# Role of Blood Gas Analysis During Cardiopulmonary Resuscitation in Out-of-hospital Cardiac Arrest Patients: Erratum

**DOI:** 10.1097/01.md.0000494760.10745.0a

**Published:** 2016-08-26

In the article “Role of blood gas analysis during cardiopulmonary resuscitation in out-of-hospital cardiac arrest patients”,^1^ which appeared in Volume 95, Issue 25 of *Medicine*, Dr. Youn-Jung Kim's surname was omitted from the byline originally. The article has since been corrected online.
